# How Do Failed Entrepreneurs Cope with Their Prior Failure When They Seek Subsequent Re-Entry into Serial Entrepreneurship? Failed Entrepreneurs’ Optimism and Defensive Pessimism and Coping Humor as a Moderator

**DOI:** 10.3390/ijerph18137021

**Published:** 2021-06-30

**Authors:** Kumju Hwang, Jinsook Choi

**Affiliations:** 1 Department of Business Administration, Chung-Ang University, Seoul 06974, Korea; kumju@cau.ac.kr; 2 Graduate School, Chung-Ang University, Seoul 06974, Korea

**Keywords:** defensive pessimism and optimism, fear of failure, career ambition, coping humor

## Abstract

Entrepreneurial failure is prevalent, and particularly when the COVID−19 crisis exacerbates the economic recession, it becomes even more prevalent. Entrepreneurs experience an intensive emotional crisis when their ventures fail, and this deleterious impact, including stress and emotional pain, may prevent failed entrepreneurs (FEs) from restarting; hence, how they cope with failure has received increased attention in recent years. However, most of the extant literature focuses on success rather than failure, and there is very limited literature on how FEs cope with the psychological and emotional crisis caused by failure. This study focuses on FEs’ use of optimism and defensive pessimism as coping strategies within the mental simulation theory with respect to their re-entry intentions. It examines the impact of career ambition and public self-awareness on optimism, of the fear of failure (FoF) and self-doubt, on defensive pessimism, and of coping humor as a moderator. We used structural equation modeling to analyze the data of 277 Korean FEs who have actual entrepreneurial failure experiences and actively prepared for their re-entry. The results show that career ambitions and public self-awareness have an impact on optimism, and FoF and self-doubt lead to defensive pessimism. Coping humor also has a moderating effect on the path from defensive pessimism to the intention to re-enter. This study advances the literature on coping mechanisms that FEs employ to manage the negative impact of failure and prepare for their subsequent re-entry. Its theoretical model, based on the mental simulation theory combined with social comparison theory, provides a possible integrative framework that includes both the pervasively held view of entrepreneurs’ optimism related to overconfidence and their defensive pessimism related to their vulnerability due to their ventures’ failure. Thus, this study makes theoretical contributions to the literature of entrepreneurial failure, as well as practical implications for policymakers and educators who assist FEs in successfully coping with entrepreneurial failure and re-entry.

## 1. Introduction

The failure of entrepreneurs is a prevalent phenomenon [1,2,3]. Since the COVID-19 crisis exacerbates the economic recession, entrepreneurial failure has become even more prevalent. When entrepreneurs fail, they experience an intensive emotional crisis that is similar to divorce or bereavement, and some of them commit suicide [4]. This crisis manifests as an actual fear of failure (FoF) that lasts even after the venture fails [4]. This deleterious impact, including stress and emotional pain, may prevent failed entrepreneurs (FEs) from restarting; hence, how they cope with failure has received increased attention in recent years [5]. Failed entrepreneurs’ re-entry is also prevalent [3]. The failure of a business can work as leverage for success, because serial entrepreneurs who recover from a prior failure tend to become resilient and have the potential to successfully run a new venture [6]. However, there are unresolved questions about who subsequently starts another business after a prior business failure, as well as whether it is fair to view those who do so as overconfident and reckless narcissists who neglect the risk of failure.

Entrepreneurs’ response to the emotional crisis caused by a venture’s failure is an aspect that has been underexplored. Specifically, there is a need to address relevant research questions, such as what coping strategies and emotional or psychological coping mechanisms they employ or how they develop emotional competence [7]. Additionally, it is important to examine the factors that drive FEs (who are aware of the risk of failure and have experienced painful psychological, emotional, financial, and social consequences that could lead some of them to attempt suicide) to engage in subsequent entrepreneurial behaviors. FEs need to make serious decisions that involve two possibilities: one is a successful re-entry that can help them regain their reputation, self-worth, and pride, which creates the desire for and intention to re-entry, and the other is the risk of repetitive failure that will exacerbate the irreparable stigma of failure, their stained reputation, and their strong negative emotions, which elicits an intensive FoF. Optimism or overconfidence, which is regarded as a trait that encourages re-entry [8], may not be enough for FEs to deal with the intensive FoF associated with re-entry, and thus, a coping strategy like defensive pessimism might be helpful. Their FoF elicits cognitive and emotional reactions to obstacles and difficulties in the re-entry process [9].

To understand FEs’ affect, cognition, and coping strategies as underlying processes in their decision-making for re-entry, it is important to investigate their re-entry intentions [10] that cognitively represent a re-entry choice as the outcome of the decision-making process [11]. FEs’ re-entry intentions require cognitive choices, made through decision-making processes [11], of serial entrepreneurial behaviors. Kruger et al. [10] emphasized that intentions that can powerfully predict entrepreneurial behaviors that entail considerable and comprehensive planning and contemplation. FEs’ decision to re-enter does not rely on a reflex [10] or an optimistic intuition, which leads us to consider a more comprehensive model for their re-entry intentions. Hence, this study examines FEs’ re-entry intentions, as well as their influencing factors.

Previous studies on entrepreneurial failure can be broadly classified into three categories: the attribution, effects, and results of entrepreneurial failure. The literature on the attribution of failure addresses the conceptualization of failure [12] and entrepreneurs’ causal ascription of failure [13,14,15,16]; the effects of failure that address emotion and cognition [17,18,19], stigma [20,21,22,23], and coping reactions and recovery from failure [14,24,25,26]; and the results of failure that address business opportunities [27], their subsequent behavior [13,28], and learning from failure [1,4,29,30,31]. However, the body of the literature on entrepreneurial failure is still in its infancy. Although the re-entry behavior and its influencing factors have received attention recently [15,32,33], studies focusing on FEs [26] and how prior failures affect re-entry have been limited [34]. Moreover, the coping strategies employed by FEs to deal with their negative emotions and the re-entry preparations have been scarcely studied. As individual entrepreneurs’ personal attributes influence their entrepreneurial behaviors more than contextual factors do [35], research on the characteristics or psychological dispositions of Fes, with an emphasis on individual entrepreneurs as key agents of entrepreneurial behavior [36], is critical to understanding their restart intentions. In contrast, most of the extant literature on serial entrepreneurs compares different types of entrepreneurs [37], and studies on serial entrepreneurship intentions have been more focused on success than failure.

This study focuses on FEs’ use of optimism and defensive pessimism as coping strategies with respect to their intentions to re-enter. It verifies an alternate comprehensive process underlying FEs’ re-entry intentions by proposing defensive pessimism and optimism as perpetual and cognitive bases for their re-entry intentions and examines the exogenous factors influencing the two bases. Additionally, it addresses the gap in research by investigating how FEs deal with FoF using defensive pessimism within the mental simulation theory. The application of this theory in relation to business failures has been under-researched. As entrepreneurial intentions can explain and predict entrepreneurial behaviors better than situational and personal variables [10], it is useful to analyze FEs’ intentions to restart a venture and the related influencing factors. The Korean society and government have been encouraging and fostering venture creations, which has led to increasing interests in starting a business in Korea [38]. However, the FoF prevents potential entrepreneurs from starting new ventures and re-entry [38]. As the Korean government’s recent focus has been shifted from the venture of the creation of potential entrepreneurs to FEs’ re-entry, it provides education programs to FEs who actively seek re-entry. It is, therefore, appropriate for us to investigate Korean FEs’ re-entry to explore FEs’ coping strategies to facilitate their re-entry. An empirical study of 277 Korean FEs was conducted. This study conceptually elaborates on defensive pessimism and optimism as the two main perceptual and cognitive bases for FEs’ re-entry decision-making while elucidating the impact of the FoF and self-doubt on defensive pessimism and of career ambition and public self-awareness on optimism. Additionally, it provides empirical evidence on them moderating the role of coping humor as a stress-buffering mechanism in the management of emotional and cognitive appraisal. Due to the stigma effect [37], it is difficult to collect data for FEs. Accordingly, there are few studies analyzing such data. Lin and Wang [37] indicated the difficulty in collecting the data of FEs, because the stigma attached to failure prevents them from being able to discuss such experiences. Most discussions on the aspects related to FEs have been theoretical, and only a few empirical studies [1] have been conducted. Hence, this study provides empirical data to enrich the extant body of research. This study can help fill a critical gap in the extant entrepreneurial intention literature and will shed new light on the psychological and emotional aspects that FEs deal with and respond to in the decision-making process of serial entrepreneurship. As FEs are potential serial entrepreneurs, this study can contribute to the existing literature on serial entrepreneurship, as well as entrepreneurial failure. This study also provides a systematic analysis of the different roles of optimism and defensive pessimism in emotion regulation and mental rehearsal for anticipatory events.

## 2. Theoretical Background: Pre-Factual and Counterfactual Mental Simulation: Optimism and Defensive Pessimism

The mental simulation theory elucidates how entrepreneurs make sense of various uncertain situations and events, learn from past failures, and anticipate the future [38]. Mental simulations are “imitative cognitive constructions of an event or series of events based on a causal sequence of successive interdependent actions” [39] (p. 537). Gaglio [39] also indicated that people deliberate on past and future events and alternatives with particularly counterfactual thinking during mental simulations. These simulations can be particularly useful to understand FEs who are seriously contemplating entrepreneurial re-entry.

Mental simulations with counterfactual thinking, as elucidated by the functional theory of counterfactual thinking, fulfil two functions: an affective function to regulate the emotional responses and cope with the uncertainty caused by re-experience and processed emotions and a preparative function to anticipate situations by creating and rehearsing strategies to achieve one’s goals [40]. Counterfactual thinking serves as a content-specific pathway that provides specific information influencing behavioral intentions [41]. As the preparative function of mental simulations is particularly employed for planning and decision-making [42], its role in the FE’s intent to re-enter might be worthy of attention.

Baron [43] found that existing entrepreneurs do not tend to think counterfactually, have no regret over past events, and can easily admit past mistakes as compared to potential entrepreneurs. However, Baron’s [43] study compared entrepreneurs with potential and non-entrepreneurs. As compared to entrepreneurs who focus on positive thinking that allows them to perceive their situations in a more favorable light [43], FEs have to make sense of their failures, eliciting a negative state of mind and FoF. Thus, it might be important to examine whether Fes’ engagement in counterfactual thinking may have an impact on decision-making for re-entry and their intentions.

There are two types of mental simulations: process simulation, which allows individuals to rehearse the step-by-step process of achieving specific goals, and outcome simulation, which makes individuals focus on the desirable outcome of their goals [44]. Although previous studies have documented the superior results of process simulation, both types of simulations can be applied to different situations, depending on the individual’s focus on high-level desirability and low-level feasibility [45].

Pre-factual thinking as a strategy refers to the mental simulations that are considered before the fact. Roese and Epstude [40] used anticipatory counterfactual and pre-factual thinking interchangeably; however, we used pre-factual thinking as anticipatory counterfactual thinking in the present study. Pre-factual thinking leads to expectancies and predictions, and counterfactual thinking (that is, mental simulations after the fact) influences what affects the individual and their motivations and performance [46]. Optimism and defensive pessimism, widely employed strategies related to pre-factual and counterfactual mental simulations [47], reflect an individual’s response when coping with risky situations or events that are prone to failure and are a threat to self-esteem [48].

Defensive pessimism as a motivated cognitive strategy is developed by conducting extensive mental simulations of processes and outcomes to prepare for the achievement of one’s goals [49]. Noremand Cantor [50] (p. 347) defined defensive pessimism as “an anticipatory strategy that involves setting low expectations prior to entering a situation, to prevent loss of self-esteem in the event of failure” (p. 347). They described the optimistic strategy as a situation in which “expectations are high at the outset, and post hoc restructuring of the situation is done when the outcome is known” (p. 347). Defensive pessimism involves the process of strategic thinking by deliberately considering the worst-case scenarios with a negative outlook [51] to perform two functions: “a self-protective goal to prepare for possible failure” and “a motivational goal to increase the effort to enhance the prospect of doing well” [46] (p. 2010). Optimism involves a retrospective strategy, while defensive pessimism incorporates an anticipatory strategy that enables individuals to control their anxiety and devise effective behaviors [52].

Optimism and defensive pessimism are coping strategies that are independent of each other, and individuals can employ them simultaneously, as they differ from the personality dispositions of pessimism and optimism [53]. Failure and expectations to re-try tend to generate upward counterfactual simulations and high dissatisfaction [54], whereas downward counterfactual simulations elicit a more positive feeling [55]. According to the social comparison theory, upward social comparisons provide appropriate self-betterment information for people who have coped with negative situations or events [56]. Further, optimism (retrospective strategies) leads to downward simulations, while defensive pessimism leads to upward simulations [56], which enable individuals to protect their self-esteem from the impact of possible failure by deliberately setting low expectations through upward comparisons. These low expectations also enable defensive pessimists to prepare for alternative outcomes in an upward direction [57].

To conclude, the simulation theory and the social comparison theory consider optimism and defensive pessimism as two differently directed simulations: optimism is a downward counterfactual mental simulation with retrospective strategies, and defensive pessimism is an upward pre-factual mental simulation with anticipatory strategies [46]. As FEs might suffer from negative emotions and stress due to previous failures, they may utilize defensive pessimism [58]. In particular, individuals tend to employ defensive pessimism in situations that entail a FoF and aspiration for success [53]. Hence, FEs might be appropriate candidates to promote defensive pessimism. However, as optimism enables FEs to regain self-esteem in the event of failure [46], they may also simultaneously promote optimism. Moreover, as counterfactual thinking is commonly activated by the failure to achieve goals rather than success and has an informational effect on the behavioral intentions related to the achievement of goals [41], optimism based on downward counterfactual simulations might be pertinent to the situations faced by FEs. Optimism, in this case, does not imply avoiding failure but provides optimistic information generated by a counterfactual simulation of prior failure. Therefore, this study explored the influence of FEs’ optimism and defensive pessimism as coping strategies within the perspective of mental simulations combined with the social comparison theory. Additionally, it investigates the factors that influence optimism and defensive pessimism.

## 3. Hypothesis Development

### 3.1. Hypotheses

With a focus on FEs, this study attempts to analyze their FoF while taking into consideration the high failure risk of new ventures [4,59]. In this respect, a FoF can be defined as “a dispositional tendency to experience apprehension and anxiety in evaluative situations because individuals have learned that failure is associated with aversive consequences” [60] (p. 273).

The negative impact of a FoF of potential entrepreneurs on the assessment of risks related to starting a new business has been documented by many scholars [61,62,63,64,65]. Most of the previous studies highlight the negative influence of entrepreneurial intentions and subsequent re-entry into an entrepreneurial career [66] by revealing that it heightens the risk attitudes of potential entrepreneurs towards launching new ventures [67]. A FoF is genuinely traumatic for FEs and plays a serious affective and cognitive role while assessing the relevant information and making decisions related to their subsequent entrepreneurial behavioral intentions. Although FoF has recently received a lot of attention, the previous research on this subject is insufficient [68], especially with regards to the influence of FoF on coping strategies embracing a specific mental simulation with a particular cognitive style.

The achievement motivation theory assumes that FoF acts as a motive for people to reduce the possibility of failure [60]. Stroe et al. [69] argued that dispositional FoF leads entrepreneurs to experience both affective and cognitive aversive consequences, and entrepreneurs with a high FoF believe that failure signifies universal incompetence and being unworthy of social appreciation. An entrepreneur’s self-image and vulnerability is closely related to the FoF, which hinders their optimism [70]. The FoF elicits specific types of self-regulatory tools, including cognitive strategies to achieve goals [71]. The FoF may drive entrepreneurs to seek possible solutions [72] and engage in entrepreneurial tasks [73]. Elliot and Church [71] indicated that a FoF and a strong desire to succeed can be considered antecedents of defensive pessimism. Thus, the FoF of FEs who pursue re-engagement in an entrepreneurial career may lead to defensive pessimism that serves to regulate their emotions and acts as an anticipatory preparative function to retry new venture creations.

**Hypothesis** **1.** *Failed Entrepreneurs’ Fear of Failure is Positively Associated with Their Defensive Pessimism.*

Self-doubt, which is an expression of one’s sense of uncertainty about one’s own competence, is a ubiquitous human trait and does not necessarily result in negative consequences, except in the case of serious chronic self-doubt [74]. It creates obstacles when combined with the demands and uncertainties associated with entrepreneurial processes [75]. Self-doubt and the associated coping strategies are of special relevance in the domain of FEs, because self-doubt manifests when the likelihood of failure increases [76]. Individuals with a heightened state of self-doubt harbor doubts about their success and are susceptible to the negative consequences of failure when facing upcoming tasks. They also tend to develop strategies to deal with the self-doubt [77].

Individuals tend to adopt defensive strategies and behaviors to cope with self-doubt [74]. People with high self-doubt are inclined to attribute their personal success or failure to their abilities [78]. They particularly view failure as a sign of poor ability [79], which elicits a negative effect with regard to re-entry. Employing defensive pessimism cushions this effect and softens the blow of potential failure [71]. The self-worth theory of motivation suggests that individuals need to protect their self-worth from being damaged by failure. This implies that their private and public perceptions of ability are commonly equated with self-worth, leading them to employ a protective strategy such as defensive pessimism [80]. Individuals who doubt their ability to successfully complete tasks experience a FoF [81] and may also employ defensive pessimism. Defensive pessimists exhibit self-doubt in their abilities; hence, self-doubt is found to be strongly correlated to defensive pessimism as a coping strategy [77].

**Hypothesis** **2.** *Failed Entrepreneurs’ Self-Doubt is Associated with Their Defensive Pessimism.*

Individuals’ entrepreneurships have been viewed within the perspective of their career choices [82]. Although entrepreneurship research has regarded entrepreneurship as an absorbing state at a final destination, contrary to a transient state, serial entrepreneurship can be viewed from a career perspective, because entrepreneurs contemplate their career with “a series of distinct entrepreneurial experiences that have beginnings and endings” [32] (p. 239). When some FEs make a decision to exit from entrepreneurship and become an employee, they no longer consider serial entrepreneurship as their career. However, other FEs who decide to continue their entrepreneurial career may have career ambitions to be a successful entrepreneur. Career ambition can be defined as the motivation to actively further one’s career [83], and the literature on vocational behaviors connects career ambitions to career advancements [84]. The self-determination theory (SDT) postulates that career ambitions make people strive to satisfy their psychological needs of competence and autonomy [85].

Optimism facilitates prompt decision-making while starting new ventures, as well as the drive to obtain the relevant skills and experiences [86]; the career ambitions of entrepreneurs may inculcate optimism in order to pursue career advancements. Smith et al. [87] found, with regards to an optimistic perspective, that resilience and the negation of women’s glass ceiling beliefs (which could diminish their career ambition) are positively associated with work engagement. Similarly, the career ambitions of FEs may be related to resilience and the denial of beliefs; this implies that, according to the optimistic perspective, prior failure does not lead to subsequent business failure. People with career ambitions use new orientations and skills related to optimism and risk-taking [88]. Emmerik et al. [89] found that career ambitions are negatively associated with career satisfaction, because ambitious individuals are, in general, dissatisfied with their current situations. Hence, as career ambitions drive people to pursue career satisfaction, FEs with career ambition also seek to further their career paths; this includes creating newer ventures. This step may encourage optimism as a coping strategy to boost their resilience and lead to a denial of the possibility of subsequent failures. Individuals with high ambitions are found to be optimistic [90]. As career ambitions are a motivation in advancing careers, FEs who earlier chose entrepreneurship as their career will employ optimism that fosters their re-entry intentions.

**Hypothesis** **3.** *Failed Entrepreneurs’ Career Ambitions are Positively Associated with Their Optimism.*

Self-awareness shifts the attention of individuals from the surrounding environment to themselves [91] and generates “a deep understanding of one’s emotions, one’s strengths and limitations, and one’s values and motives” [92] (p. 40). It drives individuals to engage in long-term goal-directed behaviors and effective self-regulation [93].

The differential self-awareness theory posits that public self-awareness, activated by the perception of being watched by others, leads to the adherence to social norms and standards [94]. It involves how others perceive an individual and focuses the attention of individuals on information obtained from others to appraise themselves and improve their behavior [95]. Public self-awareness also encourages people to create a positive impression of themselves [96].

The stigma associated with social dishonor and the loss of self-esteem or reputation [97] is attached to entrepreneurs’ business failures [15]. People with heightened public self-awareness are severely traumatized by a stained reputation and will crave to regain their positive image. Their strong association with the enterprise transfers the stigma of the failed enterprise to the entrepreneur. Regaining their reputation depends on successful re-entry [28]; therefore, FEs need to promote optimism. As public self-awareness responds to the role expectations established by others according to the differential self-awareness theory [94], individuals may feel obliged to fulfil the role of entrepreneurs, which may ultimately be the reason behind their optimism for re-entering entrepreneurship.

An entrepreneur’s business failure tends to negatively affect his or her self-view, because the most serious threat to self-esteem emanates from failure in those areas that influence an individual’s sense of self-worth [98]; for an entrepreneur, business failure deeply affects their self-worth. Optimism, combined with self-awareness, fosters persistence and higher engagement with a difficult task [98], and it is relevant when FEs display a re-entry behavior that is associated with a comprehensive, difficult, and persistent task. Nes et al. [99] found that high public self-awareness with optimism results in persistence. A heightened self-awareness leads to optimistic characteristics, such as resilience, and positive expectancies and consequences arising from self-regulation [100].

**Hypothesis** **4.** *Failed Entrepreneurs’ Public Self-Awareness is Positively Associated with Their Optimism.*

Although failure leads to negative emotions [4] and stress, it also provides valuable personal lessons. However, the experiences gained from failure seem valuable only upon re-entry [29]. Thus, research on FEs’ re-entry can shed new light on entrepreneurial failures and serial entrepreneurship. Intention is regarded as the individual’s cognitive state just prior to an overt behavior in cognitive psychology [101], and it is the most appropriate predictor of actual behaviors, though it is not a perfect predictor [11].

Recovery from failure is vital to make re-entry possible [15]. Coping strategies that enable FEs to control the negative impact of failure and perform mental rehearsals for evaluating the option of re-entry may play a critical role in the recovery stage.

The two main models of entrepreneurial intentions are Ajzen’s [102] theory of planned behavior (TPB) and Shapero and Sokol’s [103] entrepreneurial event model [37]. These two models suggest that entrepreneurial intentions are appropriate predictors of entrepreneurial behaviors [104]. According to Krueger [100], these models include two fundamental factors that influence intentions—perceived feasibility and perceived desirability. It is important to consider mental simulations to evaluate the feasibility of FEs’ re-entry and the factors that lead to the desirability of re-entry with regards to intentions. Thus, defensive pessimism and optimism as coping strategies and career ambition and public self-awareness as the driving forces can be important elements in the model to elucidate FEs’ re-entry intentions. Additionally, as congruence between entrepreneurs’ attributes and their past experiences of venture creation influence re-entry intentions [105], it may be important to explore their personal attributes relevant to prior failures, such as FoF and self-doubt.

**Hypothesis** **5.** *Failed Entrepreneurs’ Defensive Pessimism is Positively Associated with Their Re-Entry Intention.*

**Hypothesis** **6.** *Failed Entrepreneurs’ Optimism is Positively Associated with Their Re-Entry Intention.*

### 3.2. Coping Humor as a Moderator

Humor as a multifunctional coping strategy attenuates tension and anxiety by regulating emotions [106]. It has received increased attention in recent years [107]. The stress-buffering impact of humor reflects the widely held view that a sense of humor enables individuals to look at problems less seriously and cope with stress more effectively [108]. The negative effects of stress can be mitigated by humor [109]. Martin [108], who developed the Coping Humor Scale (CHS) for examining the stress-buffering effects of humor [110], argued that most measures are designed to test humor appreciation and focus on the types of humor but do not appropriately explore its stress-buffering effects. Coping humor refers to “using humor as a means of coping with stressful experiences” [110] (p. 1316). Although humor can help FEs to control stressful events, the role of humor in managing stress arising from entrepreneurial failure has been rarely studied [111]. Thus, this study focuses on assessing the stress-buffering effects of coping humor.

Entrepreneurs’ failures elicit a negative effect [4] and lead to stress [112]. Entrepreneurs tend to develop an emotional relationship with their business [113]; hence, business failures elicit entrepreneurial grief, which results in the degradation of FEs’ self-efficacy [37]. This may lead to self-doubt and a FoF, which forces FEs to employ defensive pessimism. The two main roles of defensive pessimism include emotion regulation and preparative function. Thus, coping humor, which promotes emotion and stress management, may effectively facilitate the path between defensive pessimism and re-entry intentions. Kuiper and Martin [114] argued that coping humor operates through the realistic cognitive processing of information. Accordingly, humor can help to cognitively organize and appraise information for mental simulations, which can facilitate FEs’ decision-making for re-entry. As humor provides entrepreneurs with a sense of control over stressful events, FEs feel more confident and optimistic [115]. Martin [108] indicated that coping humor is positively correlated with self-esteem, realistic cognitive appraisals, and optimism. Kuiper et al. [116] found that humor is a healthy coping mechanism that facilitates a positive perspective in order to view negative life events less threateningly and more constructively. Thus, humor encourages a positive view that allows for an evaluation of the feasibility of re-entry without stress. In other words, humor may fortify the influence of optimism on the intent to re-enter by mitigating the stress associated with re-entry [111].

**Hypothesis** **7.** *Coping Humor has a Moderating Effect on the Path from Failed Entrepreneurs’ Defensive Pessimism to Their Re-Entry Intentions.*

**Hypothesis** **8.** *Coping Humor has a Moderating Effect on the Path from Failed Entrepreneurs’ Optimism to Their Re-Entry Intentions.*

**Figure 1:**
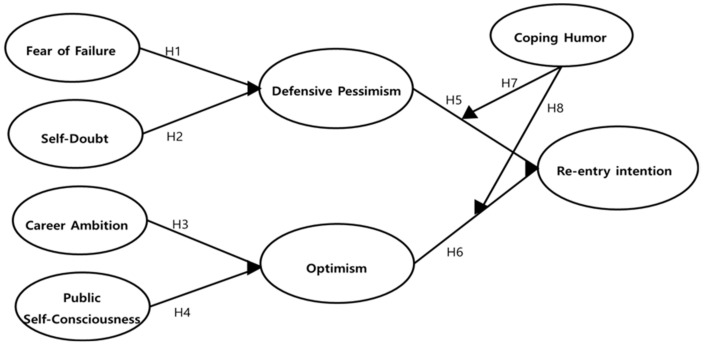
Proposed research model.

The Figure 1 presents our proposed research model.

## 4. Data and Methods

### 4.1. Data Collection and Sample

This study involved surveying FEs who are now actively seeking to re-enter entrepreneurships. The survey was conducted from 1 May 2017 to 30 April 2018, which may be regarded as a sufficient period of data collection for a single cross-sectional survey, particularly because of the unwillingness of FEs to discuss their prior experiences due to the fear of stigma. Further, the data collection period was limited to one year to reduce the influence of time-varying factors on the participants’ responses. It was difficult to find participants who met the two prerequisites: FEs whose ventures failed within the last three years and who are now actively seeking subsequent entrepreneurships. A screening question, “Did your prior venture, which has been closed for the last three years, fail?” was used. To meet the second condition, we followed judgement sampling (a non-probabilistic sampling technique). Participants were recruited from entrepreneurship education courses seeking FEs who are preparing for re-entry. Face-to-face interviews were conducted, and 332 samples were collected, of which 277 were valid.

### 4.2. Measures

To reduce the social desirability bias in the self-report survey method, we employed both procedural and statistical remedies, including the common method bias (CMB) test [117] in the robustness tests. We used verified measurement items selected from the extant literature and minimally modified them to fit the Korean context. The authors translated the measurement items into Korean and checked these with a Focus Group Interview (FGI) with five graduate students on 17 April 2017. After the first FGI, we conducted two more FGIs from 22 to 29 April 2017 with 11 current entrepreneurs who had experienced failure. The appropriateness, representativeness of the content domain, comprehensiveness, and clarity of the measurement items were evaluated through these FGIs.

All the scale items were measured on a five-point Likert scale ranging from “strongly disagree (1)” to “strongly agree (5)”. The questions for FoF were derived from Conroy et al.’s [118] five-item scale of PFAI-R (Performance Failure Appraisal Inventory-Revised), and one item was deleted because of its low inter-item correlation. The career ambition items were derived from Elchardus and Smits’s [119] five-item scale, and one item was deleted because of its low inter-item correlation. The public self-awareness items were sourced from the seven-item Self-Consciousness Scale (SCS) by Fenigstein et al. [120], and two items related to appearance, which seemed inappropriate with respect to FEs, were deleted. The defensive pessimism items were derived from the Defensive Pessimism Questionnaire (DPQ) developed by Norem [121]. We only used six DPQ items based on the adequacy of the discussions in FGIs and pretests with graduate students and entrepreneurs, because long questionnaires could cause fatigue among participants. One item of defensive pessimism was deleted because of its low inter-item correlation. The self-doubt items were sourced from the eight-item self-doubt scale by Oleson et al. [77], and four items were selected based on the suggestions garnered through FGIs and pretests with graduate students and entrepreneurs. Considering the difficulty in collecting data about FEs, it was imperative to reduce the burden on participants in order to increase the response rate [122]. Optimism was measured with the six-item scale of the psychological capital questionnaire (PCQ) by Luthans et al. [123], and two items were eliminated due to their low inter-item correlation. The re-entry intention was derived from Kickul and Zaper [124] and Schwarz et al. [125]. Coping humor items were sourced from the seven-item Coping Humor Scale (CHS) by Martin and Lefcourt [110]. The questionnaire is presented in Appendix A.

### 4.3. Data Analysis

A Cronbach’s alpha test and correlation analysis were performed using IBM SPSS 24.0 software Armonk, NY: IBM Corp. A confirmatory factor analysis (CFA) was conducted with IBM SPSS AMOS 22.0 software Armonk, NY: IBM Corp. to test the convergent validity. AMOS 22.0 was also used for analyzing the research model and hypotheses with structural equation modeling (SEM). As SEM is useful in identifying tentative cause and effect constructs, and each equation in the model displays a causal link [126], this study used SEM to analyze the causal relationships in the research model. SEM is regarded as a robust technique to test the relationships between various constructs [127].

## 5. Results

### 5.1. Demographic Characteristic Results

Table 1 presents the demographic characteristics of our respondents. A majority of the participants were male (67.15%), obtained a university degree (72.56%), and faced at least one business failure (67.87%).

### 5.2. Confirmatory Factor Analysis

Table 2 presents the CFA results of the item loadings of exogenous variables with their standard deviations, which shows the convergent validity of the measurement items. The comparative fit index CFA results indicate that the model achieved a fairly good fit to the data, minimum discrepancy per degree of freedom (CMIN/df = 1.701), root mean squared error of approximation (RMSEA = 0.050), root mean square residual (RMR = 0.048), adjusted goodness-of-fit index (AGFI = 0.847), normed fit index (NFI = 0.856), incremental fit index (IFI = 0.935), Tucker–Lewis non-normed fit index (TLI = 0.926), comparative fit index (CFI = 0.934), and goodness-of-fit index (GFI = 0.874) [128]. The RMSEA tested the fit for the global model [129], and the value of RMSEA = 0.050 in this study showed the fair model fit, because a RMSEA between 0.05 and 0.08 means a fair fit [129].

### 5.3. Results of Reliability Analysis and Correlation Matrix

According to Chin [130] and Bagozzi and Youjae [128], the adequate reliability for Cronbach’s alpha, CR (composite reliability), and AVE (average variance extracted) are 0.8, 0.7, and 0.5, respectively. As shown in Table 3, the Cronbach’s alpha of the variables was between 0.786 and 0.915; hence, both exceeded the acceptable standards. Accordingly, the scales indicated sufficient construct validity.

Although gender has a significant correlation with FoF and education has a significant correlation with FoF and career ambition, the control variables (gender, age, education, and numbers of failure experiences) do not have a significant correlation with the mediation (optimism and defensive pessimism) and dependent (re-entry intention) variables (cf. Table 4). Hence, we did not analyze the effects of the control variables further.

### 5.4. Hypotheses Testing

The overall fit of the research model was tested, and the path results are displayed in Table 5. The goodness of fit included the CMIN/df (=1.715), RMR (=0.058), GFI (=0.822), AGFI (=0.810), CFI (=0.932), NFI (=0.853), IFI (=0.933), TLI (=0.925), and RMSEA (=0.051), implying that the structural model achieved a good fit [124]. All six hypotheses were supported. Particularly, CMIN/df (=1.715), CFI (=0.932), and RMSEA (=0.051) showed a good fit [131].

### 5.5. Mediation Analysis

The mediation effect was statistically evaluated. We used a bootstrapped sampling distribution approach [132]. The analysis was performed with Hayes [133] macro model 4 and a bootstrap sample of 2000 at a 95% confidence level. Table 6 displays the results of the mediation effect analysis and the two paths (path from the FoF via defensive pessimism to re-entry intention and the path from career ambition via optimism to re-entry intention show the mediation effects).

### 5.6. Moderator Analysis

The moderating effect of coping humor on the relationship between optimism and re-entry intention and between defensive pessimism and re-entry intention was tested. We performed a multi-group analysis. It was observed that the moderating effect of coping humor existed in the relationship between defensive pessimism and re-entry intention (difference of χ^2^ = 97.085, 0.00(df = 1) > χ^2^ = 3.84, df = 1). Therefore, Hypothesis 7 is supported. The moderation test is significant when the χ^2^ value difference between constrained and unconstrained models is higher than 3.84 for one degree of freedom (df) [134]. However, a moderating effect of coping humor was not found in the relationship between optimism and the re-entry intention (difference of χ^2^ = 0.445 < χ^2^ = 3.84, df = 1). Thus, Hypothesis 8 is not supported. The Table 7 shows the results of moderating effects. For the robustness test (cf. Appendix C and Appendix D), the Hierarchical Regression for Moderation analysis indicated a moderating effect, and Appendix C and Appendix D show that a moderating effect was found only in the path from defensive pessimism to the re-entry intention. Further, Appendix E plot the moderating effects.

### 5.7. Robustness Tests

To minimize the CMB, we preserved respondent anonymity [117]; conducted FGIs, and pre-tested the survey on FEs to reduce ambiguous questions, avoid double-barreled questions [135], and ensure that the questionnaire was carefully structured [117]. Additionally, we performed robustness tests to determine the sensitivity of our results. We examined the CMB of our results using an exploratory factor analysis (EFA) with seven variables without considering a moderating variable. Our EFA results (Appendix B) showed even factors with eigenvalues >1.0, and these seven factors accounted for 68.50% of the variance. As the largest factor accounted for 10.629% of the variance, the CMB did not seem to be a major concern [117]. We also checked the highly correlated variables (r > 0.9); the highest correlation was 0.499 between optimism and career ambition, which was acceptable [136].

Our hypotheses were tested with SEM, and we performed a hierarchical regression analysis for the robustness test. Appendix E presents the results, showing that our hypotheses were supported by the SEM analysis. Additionally, the moderating effects were analyzed with a multi-group analysis, and we examined further moderating effects with the Hierarchical Regression for Moderation analysis; the results are presented in Appendix C and Appendix D.

## 6. Discussion

Drawing on the concept of coping strategies using the mental simulation theory combined with social comparison theory, we developed a research model to provide a deeper understanding of how FEs cope with prior experiences of failure when they seek subsequent re-entry. Specifically, we looked at the use of optimism and defensive pessimism as coping strategies. The integrative perspective gained from using both mental simulation theory and social comparison theory suggests that optimism and defensive pessimism are two differently directed simulations: optimism as a downward counterfactual mental simulation with retrospective strategies and defensive pessimism as an upward pre-factual mental simulation with anticipatory strategies [46].

Prior studies on entrepreneurs have mostly focused on the role of optimism but have not sufficiently researched the role of defensive pessimism in coping with the FoF. However, our study verified its role. As defensive pessimism inevitably elicits stress while anticipating future negative emotions and accepting the possibility of failure, FEs may use it reluctantly. Coping humor can help FEs to mitigate this stress and take failure less seriously during the use of defensive pessimism. Thus, our results verified the moderating role of coping humor and highlighted the importance of stress-buffering on FEs employing defensive pessimism.

Previous studies on entrepreneurial failure focused on the attribution, results, and effects of entrepreneurial failure. The literature on the attribution of failure deals with the conceptualization of failure [12] and entrepreneurs’ causal ascription of failure [13,14,15,16]. The entrepreneurial failure literature related on the effects of failure addresses emotion and cognition [17,18,19], stigma [20,21,22,23], and coping reactions and recovery from failure [14,24,25,26]. The literature on the results of failure reveals business opportunities [27], subsequent behaviors [13,28], and learning from failure [1,4,29,30,31]. However, studies focusing on FEs [26] and how prior failures affect re-entry have been limited [34]. The coping strategies employed by FEs to deal with negative emotions and the re-entry preparations have been scarcely studied. Although research on the characteristics or psychological dispositions of FEs with an emphasis on individual entrepreneurs as key agents of entrepreneurial behavior [36] is critical to understand their restart intentions, such research has been very limited. Although FEs are potential serial entrepreneurs, most studies on serial entrepreneurship intentions have been more focused on success than failure. Due to the unwillingness of FEs to discuss their prior experiences because of the fear of stigma [37], it is difficult to collect data for FEs. Hence, there are few studies analyzing such data. Lin and Wang [37] highlighted the difficulty in collecting the data of FEs. Although we could not find a large number of participants for this study, we managed to collect 277 valid data points, a reasonable amount of data, to test our model. Liu et al. [1] analyzed the valid data of only 180 FEs out of the 755 entrepreneur respondents who were approached, which emphasizes the difficulty of FE data collection due to the stigma effect.

### 6.1. Theoretical and Practical Implications

This study has several theoretical and practical implications. There are limited studies on how FEs cope with the psychological and emotional trauma caused by failure. Our study advances the literature on coping mechanisms that FEs employ to manage the negative impact of failure and prepare for their subsequent re-entry. Our theoretical model, based on the mental simulation theory combined with social comparison theory, provides a possible integrative framework that includes both the pervasively held view of entrepreneurs’ optimism related to overconfidence and their defensive pessimism related to their vulnerability due to their venture’s failure. Very few researchers have looked at the two opposing attitudes of overconfidence and vulnerability seen in FEs. Our study makes a theoretical and empirical contribution, as it deals with the two opposite, yet interrelated, psychological and emotional dispositions that FEs frequently confront. We did not regard optimism and defensive pessimism as antipodes in a set but viewed them as two independent coping strategies with different roles. Thus, the model provides an integrative framework to protect self-worth and facilitate a cognitive preparative role with mental rehearsals.

Practically speaking, educational programs conducted for FEs by the relevant government agencies, local governments, or the private sector need to promote coping strategies. The strategies should not focus only on optimism but need to develop a coordinated approach to incorporate both optimism and defensive pessimism. As discussed earlier, the survey participants attended education programs conducted by government agencies and local governments that assist FEs to effectively prepare for re-entry. After receiving the survey data from participants, we asked them if they were satisfied with the programs that they attended. Most of them were not satisfied with the programs, as the contents were similar to what they had encountered in education programs for nascent entrepreneurs. Thus, specific education contents for FEs should be developed, and coping strategies should be an important part of the education materials.

Second, this study investigates an alternate comprehensive process underlying FEs’ re-entry intentions by proposing defensive pessimism and optimism as perpetual and cognitive bases for their re-entry intentions. It helps fill a research gap in the entrepreneurial intention literature and will shed new light on the psychological and emotional aspects that FEs deal with and respond to in the decision-making process of serial entrepreneurship. The two main models of entrepreneurial intentions [37] focus on two fundamental influencing factors: perceived feasibility and perceived desirability [101]. The desire to re-enter can be augmented by various factors, and we focused on the psychological dispositions that might result from failure. The experience of failure severs entrepreneurs’ career paths and career advancement opportunities, which leads to dissatisfaction. This career-related dissatisfaction does not necessarily lead them to seek another career path but increases the desirability of re-entering entrepreneurship. Accordingly, Fes’ career ambition, which are initially tainted by frustration due to failure, tend to be heightened by their current career dissatisfaction. FEs with public self-awareness will have a growing desire to re-enter, as they are sensitive to their stained reputation and the perception that they have disappointed others because they failed to meet people’s expectations. Entrepreneurs tend to be overly optimistic about their success, and this could be the explanation for their entry even though failure rates are high [137]. The feasibility of re-entry should ideally be assessed based on their acceptance of prior and potential failure. This approach of studying feasibility based on defensive pessimism that reflects the acceptance of failure by FEs can be regarded as appropriate and novel. Thus, this study makes an important theoretical and empirical contribution by integrating failure acceptance with desirability and feasibility as the core factors influencing FEs’ intentions to re-enter entrepreneurship.

FEs should accept their failure and separate it from self-worth; hence, special programs should be designed to address these issues. Social support networks and online and offline communities for FEs can be useful for communicating and sharing their emotional and psychological trauma with community members. They will also enable FEs to utilize the learning from their past experiences for their subsequent re-entry.

Third, coping humor is particularly important for stress and emotion management of FEs in two ways: First, it enables FEs to take failure less seriously and attenuates their stress. Second, the subsequent re-entry into entrepreneurship can be a stressful event that requires difficult and complicated decision-making. FEs will also need to confront both previous failures and their fear of potential future failures. Coping humor can help them in the cognitive processing of information with regards to both failures and in organizing and appraising information for mental simulations. Accordingly, coping humor can help them in distancing themselves from experienced and expected failures. Interestingly, the results of the study indicate that coping humor fortifies the path between defensive pessimism and the intent to re-enter. The roles of coping humor and defensive pessimism (emotion regulation and preparative function) are well-matched, while coping humor might not be a suitable fit when optimism based on heightened desirability sufficiently highlights the positive aspects of re-entry. In particular, although defensive pessimism is a goal-directed positive strategy, it needs to deal with failure, which causes considerable stress and emotional pain for FEs who continuously suffer from the trauma arising from failure and the FoF. Consequently, coping humor is an effective moderator for the emotional regulation role of defensive pessimism. In practical terms, general humor therapy can act as an effective method of emotional regulation for FEs, and a focused humor therapy combined with defensive pessimism will lead to better results.

Fourth, due to the stigma effect [37], it is difficult to collect data for FEs whose immediate prior ventures have failed and who are now actively seeking subsequent entrepreneurial engagements. Our one-year data collection seems rather long for a single cross-sectional survey, particularly because of the unwillingness of FEs to discuss their prior experiences due to the fear of stigma. Hence, there are few studies analyzing such data. Lin and Wang [37] indicated the difficulty in collecting data of entrepreneurs with failure experiences, because the stigma attached to the failures prevents them from being able to discuss such experiences. Although we could not find a large number of participants for this study, we managed to collect a reasonable amount of data to test our model. Due to the stigma effect, Liu et al. [1] could analyze the valid data of only 180 entrepreneurs out of the 755 entrepreneur respondents who were approached. The present study helps in further alleviating the problem of hypotheticality related to research about FEs by analyzing real field data.

### 6.2. Limitations and Future Directions

This study can be extended in several directions. Due to the limitations of scope, we did not examine the psychological or emotional elements as influencing factors with regard to the coping strategies, and further studies may be needed to investigate these elements. For example, shame, guilt, and anger caused by failure and self-compassion or self-forgiveness might lead to different engagement behaviors with respect to re-entry.

The degree and impact of the stigma of entrepreneurial failure can vary culturally. Collectivist cultures tend to stigmatize failure to a larger extent and are less tolerable [138], and FEs in East Asian cultures feel more shameful about failure than those in Anglo cultures [139]. It would be important to analyze these cultural differences and their comparative influences on FEs’ coping and defense mechanisms.

This study primarily focused on re-entry intentions; however, further studies can make theoretical and practical contributions with investigations into the roles of various coping strategies and effects of FoF and self-doubt on re-entry and serial entrepreneurship. Further experiments or different methods investigating the various cognitive, emotional, psychological, and behavioral aspects related to the FoF and the trauma suffered by FEs may provide interesting research findings.

## 7. Conclusions

This study aimed to address the issue of how FEs cope with prior entrepreneurship failures and seek re-entry into entrepreneurship. FEs cannot avoid the feelings associated with prior failure, even with overconfidence or illusionary control; thus, they must confront these feelings. This study attempted to extend the literature on entrepreneurial failure and serial entrepreneurship by exploring how prior failures lead FEs to use particular coping strategies when they seek subsequent re-entry. We hope that this study can stimulate research on how entrepreneurial failure affects entrepreneurs’ emotions, cognition, psychology, and behaviors.

## Figures and Tables

**Table 1:** Demographic profiles of the respondents.

Characteristics		Frequency	Ratio (%)
Gender	Male	186	67.15
	Female	91	32.85
Age	20–29 years	17	6.14
	30–39 years	71	25.63
	40–49 years	100	36.10
	50–59 years	69	24.91
	60–69 years	18	6.50
	Above 70 years	2	0.72
Education Level	Elementary school	1	0.36
	Junior high school	5	1.81
	High school	66	23.83
	Bachelor’s degree	201	72.56
	Others	4	1.44
Entrepreneurship Failure Experiences	One time	188	67.87
	Two times	55	19.86
	More than three times	34	12.27
Industry	Food industry	75	24.59
	Wholesale and retail industry	44	14.43
	Service industry	98	32.13
	Manufacturing industry	37	12.13
	Technology venture industry	46	15.08
	Total	277	100.00

**Table 2:** Results of the confirmatory factor analysis.

Construct	Item	Estimate	S.E.	C.R.	*p*
Fear of Failure	FoF5	1			
	FoF4	1.007	0.071	14.243	***
	FoF2	1.052	0.076	13.845	***
	FoF1	0.742	0.064	11.636	***
Self-Doubt	Self-Doubt4	1			
	Self-Doubt3	0.828	0.048	17.146	***
	Self-Doubt2	0.938	0.041	22.97	***
	Self-Doubt1	0.856	0.044	19.422	***
Career Ambition	CA5	1			
	CA3	1.28	0.112	11.471	***
	CA2	1.142	0.113	10.098	***
	CA1	1.161	0.106	10.998	***
Public Self-Consciousness	PSC5	1			
	PSC3	1.067	0.08	13.265	***
	PSC2	0.819	0.076	10.734	***
	PSC1	0.718	0.067	10.646	***
	PSC4	0.974	0.078	12.545	***
Defensive Pessimism	DP5	1			
	DP4	1.042	0.079	13.151	***
	DP1	0.732	0.073	10.001	***
	DP2	0.185	0.064	2.906	0.004
	DP6	1.006	0.075	13.438	***
Optimism	Optimism4	1			
	Optimism3	0.894	0.066	13.496	***
	Optimism2	1.14	0.072	15.923	***
	Optimism1	1.186	0.067	17.575	***
Re-entry Intention	REI4	1			
	REI3	0.814	0.069	11.717	***
	REI2	0.926	0.067	13.894	***
	REI1	0.881	0.065	13.466	***
	REI5	0.904	0.082	10.957	***

*** *p* < 0.00, ** *p* < 0.01, and * *p* < 0.05. S.E: standard error; C.R: critical ratio.

**Table 3:** Reliability analysis.

Variable	Cronbach’s α	AVE
Fear of Failure	0.857	0.895
Self-Doubt	0.915	0.762
Career Ambition	0.828	0.761
Public Self-Awareness	0.865	0.742
Defensive Pessimism	0.786	0.845
Optimism	0.897	0.644
Re-entry Intention	0.855	0.673

**Table 4:** Correlation results.

	Data	Mean	Std.Dev	Min	Max	1	2	3	4	5	6	7	8	9	10	11
Gender	277	1.33	0.471	1	2	1										
Age	277	3.02	1.039	1	6	0.148 *	1									
Education	277	3.74	0.58	1	6	−0.115	−0.225 **	1								
Failure Experiences (number)	277	2.45	0.724	1	5	−0.107	0.141 *	−0.077	1							
Fear of Failure	277	3.5	0.907	1	5	−0.123 *	−0.093	0.170 **	0.018	**0.619**						
Self-Doubt	277	2.7	0.970	1	5	−0.035	0.082	−0.048	0.005	−0.067	**0.702**					
Career Ambition	277	4.0	0.708	2	5	−0.096	−0.031	0.142 *	−0.055	0.082	−0.112	**0.622**				
Public Self-Consciousness	277	3.4	0.773	1	5	−0.103	0.044	−0.062	−0.050	0.069	0.006	0.075	**0.588**			
Defensive Pessimism	277	3.6	0.776	1	5	−0.074	0.030	0.040	0.035	0.012	0.015	0.165 **	0.072	**0.585**		
Optimism	277	3.9	0.591	1	5	0.036	−0.042	0.117	−0.091	0.061	−0.162 **	0.499 **	0.150 *	0.136 *	**0.786**	
Re-entry Intention	277	3.8	0.596	1	5	−0.071	0.036	0.094	−0.093	0.029	−0.047	0.421 **	0.052	0.246 **	0.351 **	**0.685**

*** *p* < 0.00, ** *p* < 0.01, and * *p* < 0.05, (two-tailed). *n* = 277. Diagonal elements (in bold) are the square roots of the AVE values. Off-diagonal elements are the correlations of the variables of interest to the study. Std.Dev: standard deviation. Bold indicates that diagonal elements (in bold) are the square roots of the AVE values.

**Table 5:** Results of the path analysis.

H	Path	Path Coefficient	S.E	C.R	*p*	
H1	Fear of Failure → Defensive Pessimism	0.119	0.049	2.407	0.016 *	Supported
H2	Self-doubt → Defensive Pessimism	0.139	0.044	2.338	***	Supported
H3	Career ambition → Optimism	0.448	0.039	3.213	0.008 **	Supported
H4	Public self-consciousness → Optimism	0.392	0.046	2.070	0.044 *	Supported
H5	Defensive Pessimism → R-EI	0.380	0.065	2.176	0.003 **	Supported
H6	Optimism → R-EI	0.173	0.061	2.842	0.004 **	Supported

*** *p* < 0.00, ** *p* < 0.01, and * *p* < 0.05. R-EI: Re-entry intention.

**Table 6:** Mediation analysis.

Path	Coefficient	S.E.	*t*	*p*	LLCI	ULCI
FoF-DP-Re-Entry Intention	0.1887	0.0449	4.1992	0.0000	0.1145	0.2629
SD-DP-Re-Entry Intention	−0.0313	0.359	−0.8716	0.3842	−0.1021	0.0394
CA-O-Re-Entry Intention	0.2762	0.0523	5.2775	0.0000	0.1732	0.3792
PSC-O-Re-Entry Intention	−0.0004	0.0441	0.9920	0.2783	−0.0873	0.0864

LLCI = lower limit confidence interval, ULCI = upper limit confidence interval, FoF = Fear of Failure, DP = defensive pessimism, SD = self-doubt, O = optimism, CA = career ambition, and PSC = public self-consciousness.

**Table 7:** Moderating effect analysis.

Path	High Coping Humor			Low Coping Humor			Unconstrained Model	Constrained Model	Difference of Chi-Square
	Estimate	S.E.	C.R.	Estimate	S.E.	C.R			
DP Re-Entry Intention	0.324	0.498	2.651	0.853	0.344	2.480	210.016	294.392	97.085 ***
Optimism→Re-Entry Intention	0.283	0.090	3.152	0.376	0.098	3.821	210.016	210.461	0.445

DP: defensive pessimism, *** *p* < 0.00.

## Appendix A. Question Items

**Table A1:** Question Items.

Variables	Items
Fear of failure	When I am failing, I am afraid that I might not have enough talent. When I am failing, it upsets my “plan” for the future. When I am failing, important others are disappointed. When I am failing, I worry about what others think about me.
Self-doubt	More often than not I feel unsure of my abilities. Sometimes I feel that I don’t know why I have succeeded at something. I sometimes find myself wondering if I have the ability to succeed at important activities. I often wish that I felt more certain of my strengths and weaknesses.
Career ambition	I have a lot of plans for my professional future. I can describe myself as ambitious. Professionally, I have a number of goals I definitely want to realize. I think I will be able to realize a nice professional career.
Public self-consciousness	I’m concerned about my style of doing things. I’m concerned about the way I present myself. I usually worry about making a good impression. One of the last things I do before I leave my house is look in the mirror. I’m concerned about what other people think of me.
Optimism	When things are uncertain for me at work, I usually expect the best. I always look on the bright side of things regarding my job. I’m optimistic about what will happen to me in the future as it pertains to work. I approach this job as if “every cloud has a silver lining”.
Defensive Pessimism	I often start out expecting the worst, even though I will probably do OK. I worry about how things will turn out. I carefully consider all possible outcomes. I imagine how I would feel if things went badly. Considering what can go wrong helps me to prepare.
Re-entry	I will probably own my own business one day. It is likely that I will personally own a small business in the relatively near future. Being “my own boss” is an important goal of mine. I often think of having my own business. How likely is it that you will set up (another) business during the next two years?
Coping Humor	I often lose my sense of humor when I am having problems. I have often found that my problems have been greatly reduced when I try to find something funny in them. I usually look for something comical to say when I am in tense situations. I must admit my life would probably be a lot easier of I had more of a sense of humor. I have often felt that if I am in a situation where I have to either cry or laugh, it’s better to laugh. I can usually find something to laugh or joke about even in trying situations. It has been my experience that humor is often a very effective way of coping with problems.

## Appendix B. Robustness Test

**Table A2:** Exploratory factor analysis results.

Division		Factor Loading							Eigenvalue	Cumulative Variance
		1	2	3	4	5	6	7		
Public Self-Consciousness	PSC4	−0.842	0.083	0.076	0.078	0.021	0.037	−0.003	6.007	10.629
	PSC3	0.815	−0.016	0.016	0.061	0.071	0.081	0.043		
	PSC1	0.788	0.054	−0.006	0.003	0.036	−0.016	0.078		
	PSC2	0.788	0.016	−0.042	−0.008	−0.040	−0.006	−0.111		
	PSC5	0.779	−0.060	−0.007	0.121	0.044	0.013	0.085		
Re-Entry Intention	REI4	0.005	0.830	−0.032	0.175	−0.005	0.119	0.067	3.338	21.236
	REI1	0.019	0.815	0.031	0.099	−0.010	0.060	0.076		
	REI2	−0.001	0.807	−0.012	0.133	−0.005	0.053	0.178		
	REI3	0.095	0.733	−0.020	0.010	0.061	0.150	0.156		
	REI5	−0.047	0.664	−0.014	0.163	−0.008	0.061	0.224		
Self-Doubt	SD4	−0.024	−0.051	0.930	−0.066	−0.017	−0.015	0.008	3.265	31.692
	SD2	0.053	0.016	0.907	−0.095	−0.048	0.039	−0.037		
	SD1	−0.022	−0.026	0.872	−0.047	−0.017	−0.018	−0.041		
	SD3	0.000	−0.009	0.832	−0.070	−0.052	−0.015	−0.072		
Optimism	O1	0.081	0.149	−0.094	0.888	−0.022	−0.019	0.194	2.705	41.669
	O2	0.092	0.190	−0.036	0.827	−0.001	−0.016	0.217		
	O4	0.049	0.153	−0.060	0.816	0.098	0.108	0.174		
	O3	0.051	0.098	−0.087	0.780	0.012	0.109	0.215		
Fear of Failure	FoF5	0.076	−0.009	−0.024	0.056	0.866	0.020	−0.004	2.567	50.859
	FoF2	0.003	0.013	0.016	−0.004	0.850	0.012	0.096		
	FoF4	−0.018	0.025	−0.067	0.049	0.835	−0.039	0.096		
	FoF1	0.059	0.002	−0.026	−0.024	0.784	−0.006	−0.083		
Defensive Pessimism	DP6	0.025	0.045	0.009	0.022	−0.014	0.862	0.018	1.946	59.915
	DP4	0.000	0.064	−0.071	0.015	0.016	0.849	0.095		
	DP5	0.002	0.124	−0.009	0.018	−0.018	0.809	0.108		
	DP1	0.034	0.112	0.037	0.069	−0.032	0.730	−0.041		
	DP2	0.100	0.152	0.217	0.118	0.127	0.241	0.029		
Career Ambition	CA1	0.003	0.117	0.014	0.181	−0.049	0.089	0.822	1.408	68.498
	CA3	−0.031	0.233	−0.044	0.155	0.045	0.013	0.817		
	CA2	0.026	0.179	−0.067	0.203	0.112	0.039	0.727		
	CA5	0.120	0.197	−0.056	0.302	0.010	0.064	0.654		

Kaiser–Meyer–Olkin measure of sampling adequacy −0.797. Approx. chi-square 4717.991. Sig = 0.000. Extraction method: PCA (Principal Component Analysis). Rotation method: Varimax with a Kaiser normalization. (a) Rotation converged in 6 iterations. The grey background is normal for the display of exploratory factor analysis results.

## Appendix C. Robustness Test

**Table A3:** Hierarchical regression for the moderation analysis: the moderating effect of coping humor in the path between defensive pessimism and the re-entry intention.

		Model 1			Model 2			Model 3		
		B	BETA	*t*	B	BETA	*t*	B	BETA	*t*
Independent	DP	0.189	0.246	4.211 ***	0.180	0.234	4.071 ***	0.181	0.236	4.182 ***
Moderator	CH				0.216	0.197	3.426 ***	1.028	0.939	4.104 ***
Interaction term	DP * CH							0.224	0.766	3.347 ***
R Square		0.246			0.315			0.367		
Adjusted R Square		0.057			0.093			0.125		
R square change		0.061			0.099			0.135		
F change		0.000			0.001			0.001		

Durbin–Watson = 1.896. DP: Defensive pessimism; CH: Coping humor. * *p* < 0.5, ** *p* < 0.01, and *** *p* < 0.00. As the R^2^ increases (0.246 in model 1, 0.315 in model 2, and 0.367 in model 3) and the F change *p*-value is lower than 0.05 in all three models, it is evident that a moderating effect exists.

## Appendix D. Robustness Test

**Table A4:** Hierarchical regression for the moderation analysis: the moderating effect of coping humor on the path between optimism and the re-entry intention.

		Model 1			Model 2			Model 3		
		B	BETA	*t*	B	BETA	*t*	B	BETA	*t*
Independent	Optimism	0.302	0.351	6.207 ***	0.279	0.323	5.029 ***	1.087	1.259	4.061 ***
Moderator	CH				0.063	0.057	0.894	1.026	0.937	3.207 ***
Interaction term	Optimism * CH							0.237	1.570	3.084 ***
R Square		0.351			0.354			0.394		
Adjusted R Square		0.120			0.119			0.146		
R square change		0.123			0.125			0.126		
F change		0.000			0.372			0.002		

Durbin–Watson = 1.896. CH: Coping humor. * *p* < 0.5, ** *p* < 0.01, and *** *p* < 0.00. As the F change *p*-value is higher than 0.05 (0.372 in Model2), a moderating effect does not exist.

## Appendix E. Robustness Test

**Table A5:** Hierarchical regression results (dependent variable: re-entry intentions).

	Model 1	Model 2	Model 3
Gender	−0.106	−0.070	−0.050
Age	0.049	0.043	0.031
Education	0.098	0.030	0.021
Failure Experiences (Numbers)	−0.087 *	−0.065	−0.080
Fear of Failure		−0.005	−0.020
Self-Doubt		−0.004	−0.059
Career Ambition		0.249 ***	0.739 ***
Public Self-Awareness		0.119 *	0.141 *
Defensive Pessimism		0.278 ***	0.352 ***
Optimism		0.297 ***	0.584 **
FoF x Defensive Pessimism			0.228 **
Self-doubt x Defensive pessimism			0.112 *
Career Ambition x Optimism			0.239 **
Public Self-Awareness x Optimism			0.125 *
R Square	0.030	0.289	0.417
F	2.102	9.482	8.420

Model 1 includes the control variables. Model 2 includes the control variables, independent variables, and mediation variables (defensive pessimism and optimism). Model 3 includes the control, independent, and mediation variables and interactions between the independent and mediation variables. *** *p* < 0.00, ** *p* < 0.01, and * *p* < 0.05.
